# Adenosine A_2A_ Receptors Control Glutamatergic Synaptic Plasticity in Fast Spiking Interneurons of the Prefrontal Cortex

**DOI:** 10.3389/fphar.2018.00133

**Published:** 2018-03-20

**Authors:** Amber Kerkhofs, Paula M. Canas, A. J. Timmerman, Tim S. Heistek, Joana I. Real, Carolina Xavier, Rodrigo A. Cunha, Huibert D. Mansvelder, Samira G. Ferreira

**Affiliations:** ^1^Department of Integrative Neurophysiology, Center for Neurogenomics and Cognitive Research, VU University, Amsterdam, Netherlands; ^2^Center for Neuroscience and Cell Biology, University of Coimbra, Coimbra, Portugal; ^3^Faculty of Medicine, University of Coimbra, Coimbra, Portugal

**Keywords:** A_2A_ receptor, prefrontal cortex (PFC), synaptic plasticity, fast-spiking interneurons, adenosine, LTP and LTD, electrophysiology

## Abstract

Adenosine A_2A_ receptors (A_2A_R) are activated upon increased synaptic activity to assist in the implementation of long-term plastic changes at synapses. While it is reported that A_2A_R are involved in the control of prefrontal cortex (PFC)-dependent behavior such as working memory, reversal learning and effort-based decision making, it is not known whether A_2A_R control glutamatergic synapse plasticity within the medial PFC (mPFC). To elucidate that, we tested whether A_2A_R blockade affects long-term plasticity (LTP) of excitatory post-synaptic potentials in pyramidal neurons and fast spiking (FS) interneurons in layer 5 of the mPFC and of population spikes. Our results show that A_2A_R are enriched at mPFC synapses, where their blockade reversed the direction of plasticity at excitatory synapses onto layer 5 FS interneurons from LTP to long-term depression, while their blockade had no effect on the induction of LTP at excitatory synapses onto layer 5 pyramidal neurons. At the network level, extracellularly induced LTP of population spikes was reduced by A_2A_R blockade. The interneuron-specificity of A_2A_R in controlling glutamatergic synapse LTP may ensure that during periods of high synaptic activity, a proper excitation/inhibition balance is maintained within the mPFC.

## Introduction

The prefrontal cortex (PFC) is involved in the control of cognitive and executive functions, such as decision making, working memory, inhibitory control, attention, and behavioral flexibility (Dalley et al., 2004; Euston et al., 2013). The flexible regulation of these types of behavior makes it possible to properly respond to a changing environment (Arnsten, 2009). Such flexibility is thought to require plastic changes in the strength of synaptic connections (Kandel, 1976; Mayford et al., 2012), which is heavily dependent on the action of several neuromodulators (Pawlak et al., 2010; Bloem et al., 2014; Dembrow and Johnston, 2014).

One of the neuromodulators that can impact synaptic plasticity is adenosine, which is released in an activity-dependent fashion at synapses (Cunha et al., 1996; Wall and Dale, 2013). Its actions are mediated by a balanced activation of the inhibitory A_1_ receptors (A_1_R) and the facilitatory A_2A_ receptors (A_2A_R) (Cunha, 2008), which act predominantly on glutamatergic but also on GABAergic signaling (Shen et al., 2008; Rombo et al., 2015; Qi et al., 2017). While A_1_R control basal synaptic transmission, A_2A_R are selectively engaged in events where long-term potentiation (LTP) is induced (d’Alcantara et al., 2001; Rebola et al., 2008; Simões et al., 2016).

A_2A_R are present in the PFC (Van Dort et al., 2009; Pandolfo et al., 2013; van Aerde et al., 2013) and affect PFC-dependent behavior. Indeed, genetic elimination of A_2A_R decreases effort-based decision-making (Pardo et al., 2012), while enhancing working memory (Zhou et al., 2009; Wei et al., 2011) and reversal learning (Wei et al., 2011). Their possible pathophysiological relevance is highlighted by the ability of selective A_2A_R antagonists to attenuate working memory deficits (Horita et al., 2013; Li et al., 2015), and by the ability of caffeine, which antagonizes both adenosine receptors, to counteract cognitive behavioral deficits both in human subjects suffering from attention deficit hyperactivity disorder (ADHD; Leon, 2000) as well as in a rat model of ADHD (Pandolfo et al., 2013).

Despite the effects of A_2A_R on PFC-dependent behavior, it is not known how A_2A_R control the information flow and whether A_2A_R affect glutamatergic synaptic plasticity of information within the local PFC circuit. Therefore, we studied the impact of A_2A_R on synaptic transmission and plasticity at excitatory synapses onto pyramidal neurons and interneurons and at the network level in the medial PFC (mPFC). We found that A_2A_R are enriched at mPFC synapses, where A_2A_R blockade shifts the direction of plasticity at excitatory synapses onto layer 5 fast spiking (FS) interneurons from LTP to LTD, while it is ineffective at excitatory synapses onto layer 5 pyramidal neurons and reduces plasticity at the network level.

## Materials and Methods

### Animals

All studies were conducted in accordance with the principles and procedures outlined as “3Rs” in the guidelines of EU (210/63), FELASA, and the National Centre for the 3Rs (the ARRIVE; Kilkenny et al., 2010), and were approved by the Animal Care Committee of the Center for Neuroscience and Cell Biology (ORBEA 78/2013) or by the VU University’s Animal Experimentation Ethics Committee (DEC) and were in accordance with institutional and Dutch license procedures. Rats were housed in a temperature and humidity-controlled environment with 12 h light on/off cycles and *ad libitum* access to food and water.

### Membrane-Binding Assay

The density of A_2A_R in total membranes or synaptosomal membranes from the PFC was estimated by a radioligand-binding assay using a supramaximal concentration of the A_2A_R antagonist ^3^H-SCH58261 (6 nM; provided by E. Ongini, Schering-Plough, Milan, Italy), as described previously (Kaster et al., 2015; Viana da Silva et al., 2016). Specific binding was determined by the subtraction of non-specific binding measured in the presence of 3 μM XAC (Tocris).

### Subsynaptic Fractionation of mPFC Synaptosomes and Western Blot Analysis

To separate the extra-synaptic (non-active) zone, presynaptic active zone and post-synaptic fractions from synaptosomes, we used a fractionation method previously described in detail (Rebola et al., 2005; Canas and Cunha, 2016). The efficiency of separation is based on the segregation of different markers in the several fractions: SNAP-25 in the presynaptic active zone, PSD-95 in the post-synaptic density (PSD) and synaptophysin outside the active zone (extra-synaptic fraction). Western blot analysis was performed with an anti-A_2A_R antibody (1:500; sc-32261 from Santa Cruz Biotechnology; Santa Cruz, CA, United States), of which selectivity was confirmed by the lack of signal in A_2A_R knockout mice (Rebola et al., 2005).

### Whole-Cell-Recordings

Male Wistar rats (5–6.5 week-old) purchased from Charles River (Harlan) were decapitated, their brain was carefully removed and the mPFC was sliced in carbogen buffered (pH 7.4) ice-cold choline-based slicing solution containing (in mM): choline chloride 110, sodium ascorbate 11.6, KCl 2.5, NaH_2_PO_4_ 1.3, MgCl_2_ 7, CaCl_2_ 0.5, NaHCO_3_ 26, and glucose 10. Slices (350 μm) were kept at room temperature in aCSF oxygenated with carbogen in a holding chamber. Following recovery for at least 1 h, recordings from cells in L5 of the mPFC were made in oxygenated aCSF (flow rate of 2–3 ml/min, 32°C). Whole-cell patch-clamp recordings were made with borosilicate glass pipettes (3–6 MΩ) filled with an intracellular solution containing (in mM): K-gluconate 111, KCl 8, HEPES 10, Mg-ATP 4, K_2_ phosphocreatine 10, GTP 0.4, EGTA 0.2. Biocytin (0.2–0.5%) was added to all solutions for *post hoc* cell identification, and osmolarity was adjusted to 290–295 mOsm. Pyramidal L5 cells were visualized with differential interference contrast microscopy, selected on their large and pyramidal shape and further identified by their spike profile. FS interneurons were selected based on their small, round shape and further identified by their spike profile. During recordings, neurons were kept at a holding potential close to -70 mV. Recordings were made using MultiClamp 700 A/B amplifiers (Axon Instruments, Sunnyvale, CA, United States), with sampling at 10 kHz and low-pass filtering at 3–4 kHz. Recordings were digitized with an Axon Digidata 1440A and acquired using pClamp software (Axon). After experiments were completed, slices were stored in 4% paraformaldehyde for subsequent neuronal visualization and reconstruction as previously described (Mohan et al., 2015).

#### Spontaneous EPSCs

Spontaneous excitatory post-synaptic currents (sEPSCs) were recorded 5–10 min before and 25–30 min after drug incubation. Acquired data were stored for off-line analysis and events were detected using MiniAnalysis software. EPSC amplitude and frequency were determined and averaged over a 5-min time-course in each condition.

#### Evoked EPSCs

Excitatory post-synaptic currents (EPSCs) were evoked (eEPSCs) every 3.5 s using bipolar stimulating electrodes in glass pipettes filled with aCSF positioned 100–150 μm along the cell’s apical dendrite. Duration (0.5 ms) and amplitude (100–350 mA) of extracellular stimulation were controlled by Isoflex stimulators (A.M.P.I., Jerusalem, Israel) to generate a monosynaptic response. After recording a baseline for 5–10 min, drugs were added and the eEPSC response was recorded for another 20 min. In pyramidal neurons, 15 datapoints were determined and averaged in each condition (baseline, 5 min after incubation and 15 min after incubation). In FS cells, EPSC amplitude was averaged over a 5-min time-course in all conditions (baseline, 5 min after incubation and 15 min after incubation).

#### Long-Term Potentiation

Excitatory post-synaptic potentials (EPSPs) were evoked every 7 s (0.14 Hz) using bipolar stimulating electrodes in glass pipettes filled with aCSF positioned 100–150 μm along the cell’s apical dendrite. The duration (0.5 ms) and amplitude (100–350 μA) of extracellular stimulation were controlled by Isoflex stimulators (A.M.P.I., Jerusalem, Israel) to generate a monosynaptic response. Baseline EPSP was defined with an input/output curve, stimulating at below half maximum response. After obtaining a stable baseline of 3–5 min (30–43 EPSPs), LTP was induced within 15 min of whole-cell configuration with an unpaired theta burst stimulation (TBS) protocol (10 bursts of five pulses each at 100 Hz, repeated three times). This protocol triggered an optimal potentiated response in the cells, which was more reliable than other tested protocols such as spike timing dependent potentiation (STDP), although it was still highly variable especially in pyramidal neurons. Timing of EPSPs and the induction protocol was controlled by a Master-8 stimulator (A.M.P.I.). The slope of the initial 2 ms of the EPSP was taken as a measure of EPSP strength. The change in synaptic strength was defined as the percent change in EPSP slope 20–30 min after the TBS relative to baseline. Cell input resistance was monitored by applying a hyperpolarizing pulse at the end of each sweep (-30 pA). After LTP induction, membrane potential was returned to approximate baseline value by modest current injection. Criteria for inclusion of recordings were: (1) baseline resting membrane potential <-60 mV, (2) smooth rise of EPSP and clear separation from stimulation artifact, (3) stable baseline EPSP slope, (4) less than 30% change in input resistance, (5) no AP-firing evoked by extracellular stimulation in post-pairing period. In total, five cases of extreme EPSP rundown (slope < 20% of baseline) were excluded from analysis.

### Extracellular Recordings

Male Wistar rats (6–8 week-old) were purchased from Charles River Laboratories (Barcelona, Spain). Rats were anesthetized under halothane atmosphere, decapitated and the brain rapidly removed from the skull and submerged in ice-cold artificial cerebrospinal fluid (aCSF) solution of the following composition, in mM: NaCl 125, KCl 3, MgSO_4_ 1, CaCl_2_ 2, Na_2_HPO_4_ 1.25 NaHCO_3_ 25–26 and glucose 11, pH 7.4 (osmolality, ∼300 mOsmol.kg^-1^), oxygenated with carbogen (95% O_2_ + 5% CO_2_). Coronal slices (300 μm-thick) containing the medial prefrontal cortex (mPFC) were cut with a Vibratome 1500 sectioning system (Vibratome, Germany). The slices were then transferred to a pre-chamber containing aCSF under continuous oxygenation to recover at 32°C for at least 1 h. Slices were then transferred to a submerged recording chamber where they were continuously superfused at a rate of 2–3 ml/min with oxygenated aCSF at 30–32°C. A bipolar concentric stimulation electrode SNE-100 (Kopf, Germany) was placed on the layer II/III of the mPFC delivering rectangular pulses (80–160 μA) of 0.1 ms duration applied with a Digitimer DS3 stimulator (Digitimer, Ltd., United Kingdom) once every 20 s. The evoked population spikes were recorded through an extracellular borosilicate microelectrode (filled with 4 M NaCl, 2–4 MΩ resistance) placed in the layer V of the mPFC, using an Axopatch 200B amplifier (Axon Instruments, Inc., United States), coupled to an analog/digital acquisition board (Digidata 1322A; Axon Instruments, Inc., United States). Responses were digitized at 10 kHz and continuously monitored on a personal computer via WinLTP 1.1 software (Anderson and Collingridge, 2007). Responses were quantified as the amplitude of the population spike recordings. After stabilizing the response, the input/output curve was obtained. Then the intensity of the stimulus was regulated to obtain 40–50% of the maximum response before induction of LTP. LTP was induced by delivering a train of 100 Hz (50 pulses, 0.5 s duration) for a priming effect, which was 15 min later followed by four trains of 100 Hz (50 pulses, 0.5 s duration, 1 every 10 s). Due to difficulties in inducing LTP in rat PFC slices, LTP protocols were extensively tested and this protocol, which has been used by Gemperle et al. (2003), was the most reliable one in our hands.

### Experimental Design and Statistics

For membrane binding assays, PFC from five adult male Wistar rats were used, and the density of A_2A_R in synaptosomal membranes was compared to that in total membranes using unpaired *t*-test. For Western blotting of sub-synaptic fractions, we pooled together mPFC tissue from 22 rats (30–45 days-old). This was due to the requirement of 1 g of tissue for the sub-synaptic fractionation step. For pharmacology in electrophysiology experiments, all the drugs used were dissolved in aCSF at the desired concentration and bath applied during the experiments. The drugs were diluted from stock solutions made in dimethylsulfoxide (DMSO) to their final concentrations: SCH58261 (50–100 nM from 5 mM stock solution, Tocris). All experiments were performed without application of synaptic blockers. In extracellular recordings, due to high variability in LTP magnitude, whenever SCH58261 was tested, a control slice was also done in parallel. In the end, data from 25 slices per group (from 25 different rats) were pooled together for statistical comparison using an unpaired *t*-test. For plasticity in whole-cell patch-clamp experiments, due to cellular variability, strict exclusion criteria (see above), long duration of the experiment and high quality of slices needed, more animals were needed than are presented in our figures. When relevant and possible, we recorded one cell in control and one cell in drug condition from every animal. For the pyramidal-TBS experiment, 51 animals were used. For the FS interneuron-TBS experiment, 27 animals were used. Raw data was analyzed using Clampfit 10.4 and custom Matlab scripts. For all LTP experiments, we used the percentage of increase in EPSP slope (whole-cell recordings) or population spike amplitude (extracellular recordings) induced by the LTP protocol per cell (whole-cell recordings) or per slice (extracellular recordings) as input for statistical tests. An unpaired *t*-test was used to compare two groups consisting of multiple such experiments, comparing the percentage of LTP induction in control experiments *versus* the percentage of LTP induction in A_2A_R antagonist-treated experiments. This method for comparing differences in LTP induction in control *versus* drug treated slices is adopted from our previous research on LTP induction (Mansvelder and McGehee, 2000; Couey et al., 2007; Meredith et al., 2007; Rebola et al., 2008; Verhoog et al., 2013, 2016; Simões et al., 2016). For whole-cell spontaneous recordings, 12 slices from 10 different animals were used in sEPSC on pyramidal neurons; 2 recordings were excluded for rundown reasons (>20% change in resistance). For sEPSC on FS interneurons, 32 recordings of interneurons were made from slices of 21 different animals. Of these, 13 cells were actual FS interneurons; 1 was excluded for rundown. For eEPCS experiments on pyramidal neurons, 14 slices were used from four different animals. For eEPSC experiments on FS interneurons, eight slices from three animals were used, four animals were used in total for these experiments. All recorded sEPSCs were analyzed with MiniAnalysis software (Synaptosoft, version 6.0.7). All the statistical analysis was performed using Prism 6 (GraphPad software). Data was analyzed by using the appropriate parametric statistical test as mentioned in the text and *p* < 0.05 was taken as statistically significant.

## Results

### A_2A_R Are Enriched in Synaptosomal Membranes and Present in All Sub-synaptic Fractions

**FIGURE 1:**
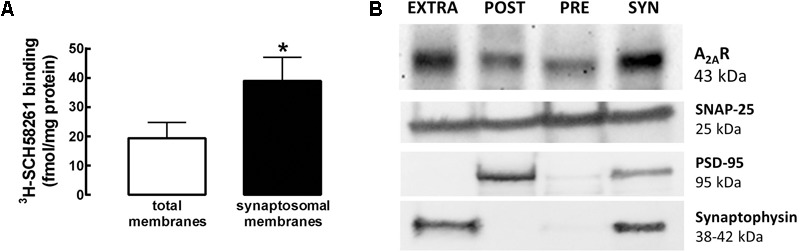
A_2A_R are enriched in synaptosomal membranes and present in all subsynaptic fractions. **(A)** The binding density of a supra-maximal concentration of a selective A_2A_R antagonist ^3^H-SCH58261 (6 nM) was higher in synaptosomal when compared to total membranes from Wistar rat PFC. Data are mean ± SEM of five rats; ^∗^*p* < 0.05, unpaired Student’s *t-*test. **(B)** Western blots of subsynaptic fractions showing the subsynaptic distribution of A_2A_R in the mPFC (pooled from 22 rats due to the requirement of large sample size – 1 g of tissue – at the start of the subsynaptic fractionation procedure). The efficiency of separation is based on the segregation of different markers in the several fractions: SNAP-25 in the presynaptic active zone, PSD-95 in the post-synaptic density (PSD) and synaptophysin outside the active zone (extrasynaptic fraction). A_2A_R are present in all the subsynaptic fractions, inside and outside the presynaptic active zone and PSD. However, there is an enrichment outside the presynaptic active zone and PSD.

To investigate the density and synaptic distribution of A_2A_R, we compared the binding of ^3^H-SCH58261 in total and synaptosomal membranes from the PFC. The binding density of ^3^H-SCH58261 was higher (*n* = 5; *t*_8_= 4.56; *p* = 0.0018; unpaired *t*-test) in the synaptosomal membrane fraction (39.0 ± 3.6 fmol/mg protein) compared to the total membrane fraction (19.4 ± 2.4 fmol/mg protein; *n* = 5) from the PFC (**Figure 1A**). Given this enrichment of A_2A_R in synaptosomal membranes, we used mPFC synaptosomes (pooled from 22 rats) to separate the different subsynaptic fractions, and probed for the subsynaptic distribution of A_2A_R. A_2A_R were present in all the subsynaptic fractions, inside and outside the presynaptic active zone and PSD, with a higher A_2A_R density observed outside the presynaptic active zone and PSD (**Figure 1B**). The presence of A_2A_R in all PFC sub-synaptic fractions suggests a role for A_2A_R in the control of synaptic communication in the mPFC.

### A_2A_R Do Not Control Spontaneous and Evoked Excitatory Synaptic Transmission

**FIGURE 2:**
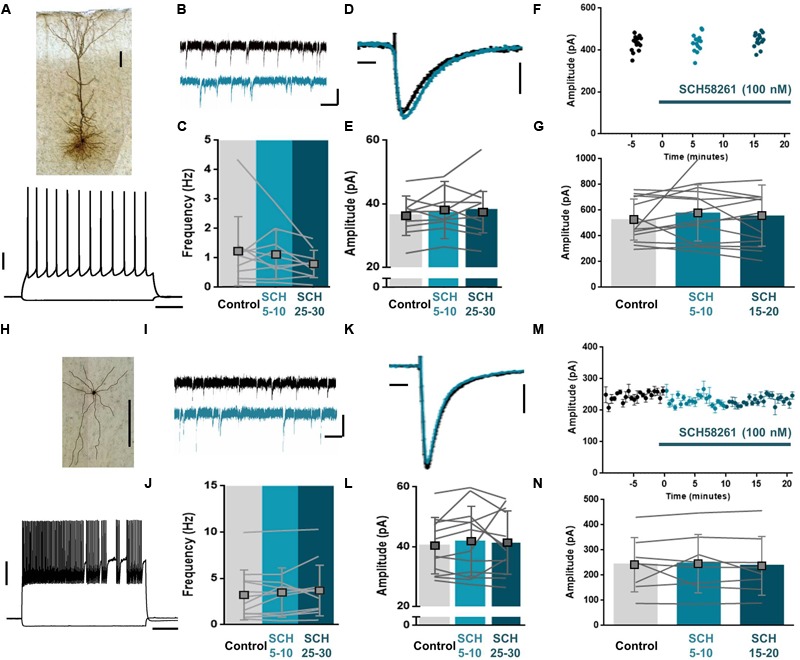
A_2A_R do not affect spontaneous or evoked excitatory synaptic transmission in the mPFC. Biocytin staining of layer 5 pyramidal neuron (**A**, top) and fast spiking (FS) interneuron (**H**, top) from coronal slice of rat prelimbic mPFC. Scale bars: 100 μm. Voltage responses to hyperpolarizing (–50 pA) and depolarizing (+225 pA) somatic current injections to the soma of a L5 pyramidal neuron (**A**, bottom) and to hyperpolarizing (–60 pA) and depolarizing (+400 pA) somatic current injections to the soma of a FS interneuron (**H**, bottom). Scale bars: 20 mV, 200 ms. **(B,C)** Bath application of the selective A_2A_R antagonist, SCH58261 (100 nM, blue) did not affect the frequency of sEPSCs onto pyramidal neurons. A representative trace is depicted in control (black) and prolonged SCH58261 (100 nM, blue) wash-in condition **(B)**. The frequency over 5 min does not change after SCH58261 (100 nM, blue) wash-in; data are mean ± SD of *n* = 10, individual lines show the average of one cell over the course of 5 min per condition **(C)**. Scale bars: 30 pA, 50 ms. **(D,E)** Bath application of SCH58261 (100 nM, blue) did not affect the amplitude of sEPSCs onto pyramidal neurons. The average sEPSC amplitude of a representative cell is depicted in control (black) and prolonged SCH58261 (100 nM, blue) wash-in condition **(D)**. The average amplitude over 5 min does not change after SCH58261 (100 nM, blue) wash-in; data are mean ± SD of *n* = 10 **(E)**. Scale bars: 10 pA, 1 ms. **(F,G)** Bath application of SCH58261 (100 nM, blue) did not affect the amplitude of eEPSCs onto pyramidal neurons. The eEPSC amplitude of a representative cell is depicted during 15 sweeps in all three conditions; control (black), short- (light blue) and prolonged SCH58261 (100 nM, blue) wash-in conditions are shown **(F)**. The average amplitude does not change after SCH58261 (100 nM, blue) wash-in; data are mean ± SD of *n* = 14 **(G)**. **(I,J)** Bath application of SCH58261 (100 nM, blue) did not affect the frequency of sEPSCs onto interneurons. A representative trace is depicted in control (black) and prolonged SCH58261 (100 nM, blue) wash-in condition **(I)**. The frequency over 5 min does not change after SCH58261 (100 nM, blue) wash-in; data are mean ± SD of *n* = 12, individual lines show the average of one cell over the course of 5 min per condition **(J)**. Scale bars: 30 pA, 50 ms. **(K,L)** Bath application of SCH58261 (100 nM, blue) did not affect the amplitude of sEPSCs onto interneurons. The average sEPSC amplitude of a representative cell is depicted in control (black) and prolonged SCH58261 (100 nM, blue) wash-in condition **(K)**. The average amplitude over 5 min does not change after SCH58261 (100 nM, blue) wash-in; data are mean ± SD of *n* = 12 **(L)**. Scale bars: 10 pA, 1 ms. **(M,N)** Bath application of SCH58261 (100 nM, blue) did not affect the amplitude of eEPSCs onto pyramidal neurons. The average eEPSC amplitude of a representative cell is depicted over a time-course of 30 min, in which control (black), short- and prolonged SCH58261 (100 nM, blue) wash-in conditions are shown **(M)**. The average amplitude over 5 min does not change after SCH58261 (100 nM, blue) wash-in; data are mean ± SD of *n* = 8 **(N)**. Paired one-way ANOVA; all data are non-significant (*p* > 0.05).

To determine whether sEPSCs or eEPSCs are affected by A_2A_R, we recorded sEPSCs and eEPSCs onto both pyramidal neurons and FS interneurons, the two largest groups of neurons in the PFC (Markram et al., 2004) and tested the effect of the selective A_2A_R antagonist SCH58261. After recording a baseline in ACSF, SCH58261 (100 nM) was incubated into the bath and cells were recorded for another 20–30 min. Spontaneous events onto pyramidal neurons were unaffected by incubation of SCH58261 (100 nM; **Figures 2A–E**) in both frequency (**Figures 2B,C**; Frequency mean control: 1.22 ± 1.2 Hz, *n* = 10; 5 min after SCH58261: 1.12 ± 0.83 Hz, *n* = 10; 25 min after SCH58261: 0.78 ± 0.46 Hz, *n* = 10; difference: *F*_2,9_= 6.05, *p* = 0.21, ANOVA) and amplitude (**Figures 2D,E**; Amplitude mean control: 36.38 ± 6.2 pA, *n* = 10; 5 min after SCH58261: 38.18 ± 8.9 pA, *n* = 10; 25 min after SCH58261: 37.50 ± 6.5 pA, *n* = 10; difference: *F*_2,9_= 0.95, *p* = 0.39, ANOVA). Similarly, spontaneous events onto FS interneurons were unaffected by incubation of SCH58261 (100 nM; **Figures 2H–L**) in both frequency (**Figures 2I,J**; Frequency mean control: 3.25 ± 2.7 Hz, *n* = 12; 5 min after SCH58261: 3.50 ± 2.64 Hz, *n* = 12; 25 min after SCH58261: 3.72 ± 2.7 Hz, *n* = 12; difference: *F*_2,11_= 1.52, *p* = 0.24, ANOVA) and amplitude (**Figures 2K,L**; Amplitude mean control: 40.34 ± 9.3 pA, *n* = 12; 5 min after SCH58261: 41.83 ± 11.5 pA, *n* = 12; 25 min after SCH58261: 41.34 ± 10.5 pA, *n* = 12; difference: *F*_2,11_= 0.32, *p* = 0.65, ANOVA). Also, eEPSCs onto both pyramidal neurons (**Figures 2F,G**) and interneurons (**Figures 2M,N**) were unaffected by incubation of SCH58261. Specifically, the amplitude of eEPSCs onto pyramidal neurons did not differ between baseline and incubation conditions (**Figures 2F,G**; Amplitude mean control: 522.6 ± 160.4 pA, *n* = 14; 5 min after SCH58261: 574.7 ± 218.9 pA, *n* = 14; 15 min after SCH58261: 554.1 ± 239.1 pA, *n* = 14; difference: *F*_2,13_= 0.67, *p* = 0.46, ANOVA) and likewise, the amplitude of eEPSCs onto FS interneurons did not differ between baseline and incubation conditions (**Figures 2M,N**; Amplitude mean control: 242.5 ± 107.3 pA, *n* = 8; 5 min after SCH58261: 246.5 ± 115.4 pA, *n* = 8; 15 min after SCH58261: 237.9 ± 116.1 pA, *n* = 8; difference: *F*_2,7_= 0.14, *p* = 0.79, ANOVA). Thus, A_2A_R do not seem necessary for excitatory synaptic transmission in the mPFC as their blockade does not affect either sEPSCs or eEPSCs in pyramidal neurons and FS interneurons.

### A_2A_R Blockade Does Not Affect Glutamatergic Synapse LTP in Layer 5 Pyramidal Neurons

**FIGURE 3:**
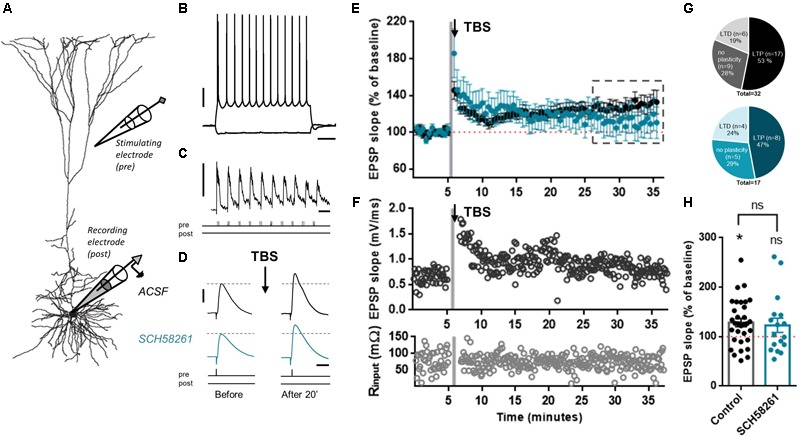
A_2A_R blockade does not affect glutamatergic synapse LTP in layer 5 pyramidal neurons. **(A)** Biocytin reconstruction of layer 5 pyramidal neuron from coronal slice of rat prelimbic mPFC showing relative positions of recording and stimulating electrodes. **(B)** Voltage responses to hyperpolarizing (–60 pA) and depolarizing (+380 pA) somatic current injections to the soma of a L5 pyramidal neuron. Scale bars: 20 mV, 200 ms. **(C)** Plasticity induction protocol. Theta burst stimulation (TBS) was induced by stimulation of 10 bursts of five pulses each at 100 Hz, repeated three times. Scale bars: 20 mV, 200 ms. **(D)** After obtaining a baseline measure of EPSPs, TBS-LTP was induced. EPSPs were then recorded for up to 30 min to observe changes in EPSP slope. Slices were pre-incubated in either control ACSF or in ACSF with added SCH58261 (100 nM). Scale bars: 2 mV, 20 ms. **(E)** Summary time-course plot of control (black symbols) and SCH58261 (100 nM; blue symbols) experiments, showing a robust LTP in control condition, and a highly variable LTP in SCH58261 pre-incubated cells. **(F)** Example of a TBS-LTP experiment in control conditions showing slope and input resistance (top and bottom panels, respectively) *versus* time. Gray shading indicates time of TBS induction. **(G)** The fraction of cells that display LTP is slightly higher in the control condition than in the presence of SCH58261; however, fraction differences were not significant (Chi-square test, *p* = 0.52). **(H)** Summary bar chart of control and SCH58261 (100 nM) TBS-LTP experiments, showing percentage change of EPSP slope for both conditions (mean ± SEM; control: *n* = 32, SCH58261: *n* = 17). Unpaired *t*-test *p* = 0.73; ^∗^*p* < 0.05 compared to baseline value of 100%, one-sample *t*-test.

Under endogenous levels of adenosine, A_2A_R mainly act as a modulator of processes in which plasticity is engaged (d’Alcantara et al., 2001; Rebola et al., 2008; Costenla et al., 2011). Therefore, we tested whether A_2A_R blockade affected the induction of glutamatergic synaptic plasticity in mPFC pyramidal neurons. We made whole-cell recordings from L5 pyramidal neurons (**Figures 3A,B**) and glutamatergic EPSPs were evoked by extracellular stimulation. To induce LTP, a TBS protocol was applied (Larson and Munkácsy, 2015). After recording a stable baseline of EPSPs, 10 bursts of five pulses each were given at 100 Hz (**Figure 3C**), and this was repeated three times within 30 s. Following this induction protocol, the slope of EPSPs was increased in a sustained manner, 20–30 min after the induction protocol (128.2 ± 46.6%, *n* = 32; **Figures 3D–F,H**). When slices were pre-incubated with SCH58261 (100 nM), the increase in EPSP slope (122.8 ± 59.8%, *n* = 17; **Figures 3D–F,H**) was not significantly different from control experiments without SCH58261 (*t*_47_= 0.35, *p* = 0.731, unpaired *t*-test). Indeed, in the absence of SCH58261, 53% of cells showed TBS-induced LTP (17 out of 32), 28% did not show a change in EPSP slope (9 out of 32) and 19% showed a reduction in EPSP slope (6 out of 32), while in the presence of SCH58261 (100 nM), 47% of the pyramidal cells showed TBS-induced LTP (8 out of 17), 29% showed no change (5 out of 17), and 24% showed a reduction in EPSP slope (4 out of 17). These distributions were not significantly different between control and SCH58261 conditions (**Figure 3G**, chi-square test, *p* = 0.52). In both conditions – control and presence of SCH58261 – cells had on average similar resting membrane potential and input resistance (mean RMP of control: -67.6 ± 0.4 mV; mean RMP with SCH58261: -69.1 ± 0.7 mV, *t*_49_= 1.82, *p* = 0.07, unpaired *t*-test; mean R input of control: 52.3 ± 4.7 mΩ; mean R input with SCH58261: 54.4 ± 7.6 mΩ, *t*_49_= 0.26, *p* = 0.80, unpaired *t-*test). Thus, blockade of A_2A_R has no significant effect on the induction of glutamatergic synaptic plasticity in L5 pyramidal neurons in mPFC slices.

### A_2A_R Blockade Shifts the Direction of Plasticity From LTP Into LTD at Excitatory Synapses Onto Layer 5 Fast Spiking (FS) Interneurons

**FIGURE 4:**
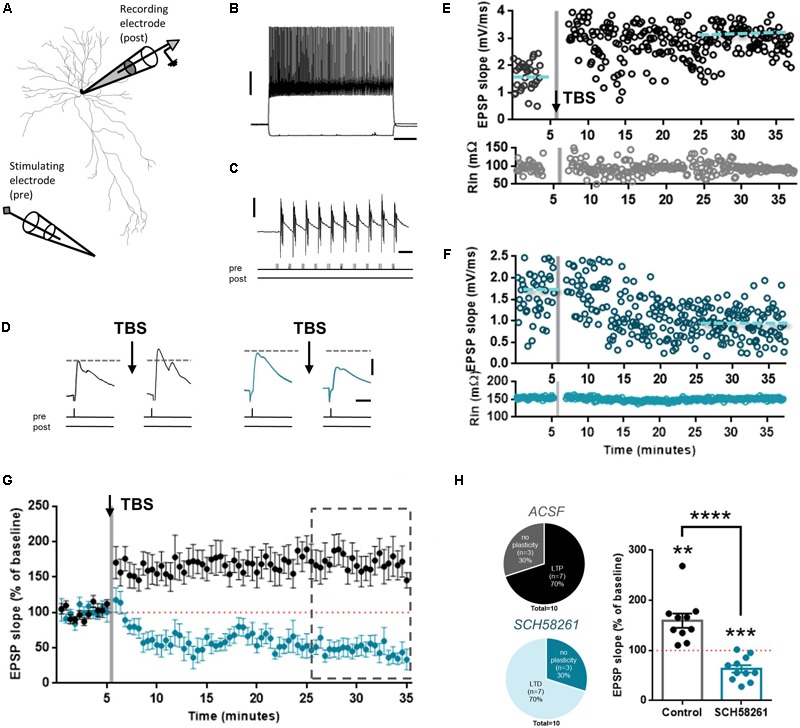
A_2A_R blockade shifts reverses LTP to LTD at excitatory synapses in layer 5 FS interneurons. **(A)** Biocytin reconstruction of a FS interneuron from coronal slice of rat mPFC showing relative positions of recording and stimulating electrodes. **(B)** Voltage responses to hyperpolarizing (–80 pA) and depolarizing (+360 pA) somatic current injections to the soma of a FS interneuron. Scale bars: 20 mV, 200 ms. **(C)** Plasticity induction protocol. TBS was induced by stimulation of 10 bursts of five pulses each at 100 Hz, repeated three times. Scale bars: 10 mV, 200 ms. **(D)** After obtaining a baseline measure of EPSPs, TBS-LTP was induced. EPSPs were then recorded for up to 30 min to observe changes in EPSP slope. Slices were pre-incubated in either control ACSF (black traces) or in ACSF with added SCH58261 (100 nM; blue traces). Scale bars: 2 mV, 20 ms. Representative TBS-LTP experiments in control **(E)** and 100 nM SCH58261 **(F)** conditions showing slope and input resistance (top and bottom panels, respectively) *versus* time. Gray shading indicates time of TBS induction. **(G)** Summary plot of control (black symbols) and SCH58261 (100 nM; blue symbols) experiments, showing a robust LTP in control condition, and a strong LTD in SCH58261 pre-incubated cells. (**H**, left panel) The fraction of cells that obtain plasticity is reversed in control *versus* SCH58261 conditions. In control, 70% of cells display LTP, whereas in SCH58261, 70% of cells display LTD. (**H**, right panel) Summary bar chart of control and SCH58261 (100 nM) TBS-LTP experiments, showing percentage change in EPSP slope for both conditions (mean ± SEM; control: *n* = 10; SCH58261: *n* = 10). ^∗∗∗∗^*p* < 0.0001 compared to the respective control (*black dots)*, unpaired Student’s *t-*test. ^∗∗^*p* < 0.01 compared to the hypothetical value of 100, one-sample *t*-test. ^∗∗∗^*p* < 0.001 compared to the hypothetical value of 100, one-sample *t*-test.

Next, we tested the effects of A_2A_R blockade on glutamatergic synaptic plasticity in FS interneurons. Glutamatergic synapses on FS interneurons can undergo LTP, albeit through different mechanisms than pyramidal neurons (Lamsa et al., 2007; Lu et al., 2007; Sarihi et al., 2008; Nissen et al., 2010; Sambandan et al., 2010; Huang et al., 2013). To test whether A_2A_R are involved in this type of plasticity, we made whole-cell recordings from mPFC L5 FS interneurons (**Figure 4A**). These neurons had FS patterns, short action potential half widths, showed no inter-spike interval adaptation, and displayed fast hyperpolarization time constants and minimal hyperpolarization amplitude (**Figure 4B**). To induce LTP, we applied the same TBS protocol as in the pyramidal neuron recordings (**Figure 4C**). This induced a robust potentiation of EPSP slope in FS interneurons (159.4 ± 44.9%, *n* = 10; **Figures 4D–G,H**). When slices were pre-incubated with SCH58261 (100 nM), stimulation with the TBS protocol induced long-term depression (LTD), rather than potentiation (64.4 ± 25.2%, *n* = 10; **Figures 4D–G,H**), which was significantly different from control (*t*_18_= 5.84, *p* < 0.0001, unpaired *t*-test). In the two conditions – absence or presence of SCH58261 – the resting membrane potential and input resistance were similar (mean RMP of control: -71.2 ± 0.9 mV; mean RMP with SCH58261: -69.6 ± 1.9 mV, *t*_18_= 0.75, *p* = 0.46, unpaired *t*-test; mean R input of control: 164.3 ± 17.8 mΩ; mean R input with SCH58261: 160.2 ± 19.2 mΩ, *t*_18_= 0.15, *p* = 0.88, unpaired *t*-test). In the absence of SCH58261, 70% of all cells displayed LTP, compared to 0% in the SCH58261 group. Conversely, 70% of all cells displayed LTD in the SCH58261 group, whereas none of the cells in the control condition displayed LTD (**Figure 4G**). This shows that A_2A_R control the direction of plasticity at glutamatergic synapses onto FS interneurons in the mPFC.

### A_2A_R Control LTP of Population Spikes in the Layer V mPFC (mPFC)

**FIGURE 5:**
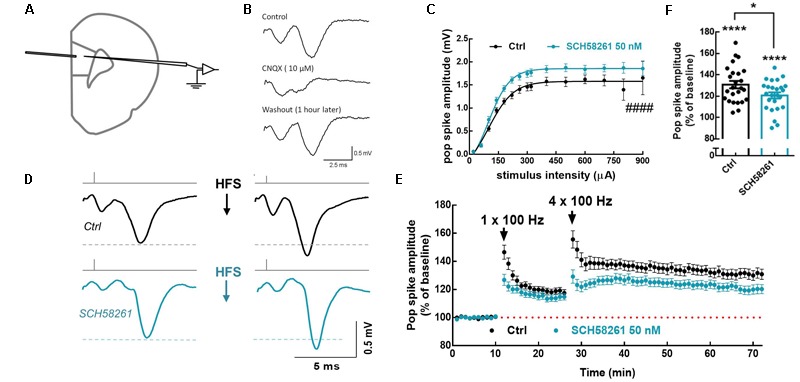
A_2A_R facilitate LTP of population spikes in mPFC layer 5. **(A)** Positioning of the stimulating (layer 2/3 mPFC) and recording electrodes (layer 5 mPFC). **(B)** The recorded population spikes were abolished by the AMPA/kainate receptor antagonist, CNQX 10 μM. **(C)** Bath application of the selective A_2A_R antagonist, SCH58261 (50 nM; blue dots) increased the number and synchrony of cells discharging action potentials as indicated by the increase in the maximum response when the input/output response was assessed 20 min after SCH58261 superfusion. **(D)** Representative averaged traces at baseline and 30 min after the induction of LTP, in the absence and presence of SCH58261 (50 nM); SCH58261 decreased LTP magnitude. **(E)** Time course showing that SCH58261 decreased the magnitude of LTP of the population spike responses triggered by a priming train of high-frequency stimulation (HFS), followed 15 min later by four HFS trains (50 pulses at 100 Hz, 0.5 s duration, delivered every 10 s). **(F)** Summary plot displaying the variability of LTP magnitude, estimated 30 min after the induction of LTP, in the absence and presence of SCH58261 (50 nM). Data are mean ± SEM of 25 slices (from 25 rats) per group. ^####^*p* < 0.0001 comparing the estimated maximum response in the presence of SCH58261 to control (*black bar/dots)*, unpaired Student’s *t-*test. ^∗^*p* < 0.05 comparing SCH58261 to control (*black bar/dots*), unpaired Student’s *t-*test. ^∗∗∗∗^*p* < 0.0001 compared to the hypothetical value of 100%, one-sample *t*-test.

Since population spikes represent the integrated responses of all local cells, i.e., responses from both pyramidal cells and interneurons, we next recorded population spikes to determine whether A_2A_R affect plasticity on the neuronal network level. To that end, we recorded extracellularly evoked AMPA receptor-mediated population spikes in mPFC layer 5 (L5) in acute brain slices, upon stimulation of L2/3 (**Figures 5A,B**). Bath application of SCH58261 (50 nM) affected the stimulus–response relationship of the network by increasing the maximum amplitude of population spikes (1.86 ± 0.03 mV in SCH58261, *n* = 23; 1.58 ± 0.04 mV in control, *n* = 26; *t*_47_= 5.34, *p* < 0.0001, unpaired *t*-test; **Figure 5C**). LTP of the population spike was induced by applying a single train of high-frequency stimulation (HFS), followed 15 min later by four HFS trains (50 pulses at 100 Hz, 0.5 s duration, delivered every 10 s). This induction protocol was run in the absence or presence of SCH58261, and for each experiment a naïve mPFC slice was used. Blockade of A_2A_R by SCH58261 decreased the magnitude of population spike LTP (120.7 ± 2.9% in SCH58261, *n* = 25; 130.9 ± 3.4% in control slices, *n* = 25; *t*_48_= 2.28, *p* = 0.027, unpaired *t*-test; **Figures 5D–F**). These findings show that A_2A_R control plasticity at a neuronal network level in mPFC.

## Discussion

In the present study, we show that in the mPFC, A_2A_R control LTP at excitatory synapses onto fast-spiking interneurons rather than onto pyramidal neurons. A_2A_R did not affect spontaneous or evoked synaptic transmission in either cell type. A similar predominant role of A_2A_R on plasticity has been observed in other brain areas, including hippocampus (Rebola et al., 2008), amygdala (Simões et al., 2016), and striatum (d’Alcantara et al., 2001; Shen et al., 2008; Li et al., 2015). As in the hippocampus (Rebola et al., 2005), A_2A_R in the mPFC are enriched at synapses. However, mPFC A_2A_R are enriched outside the presynaptic active zone and PSD, whereas most of the hippocampal A_2A_R are located inside the presynaptic active zone and PSD (Rebola et al., 2005). It is conceivable that this different sub-synaptic distribution could translate into A_2A_R playing by different rules to control information flow within the mPFC. Indeed, our results show an effect of A_2A_R antagonism on the induction of LTP at excitatory synapses specifically in FS interneurons, while the antagonist was ineffective at excitatory synapses onto pyramidal neurons. At excitatory connections to FS interneurons, the blockade of A_2A_R led to LTD of their excitatory synapses, meaning that without active A_2A_R, LTD would occur at these glutamatergic synapses onto FS interneurons. Thus, A_2A_R activation would be particularly important for the induction of synaptic potentiation of glutamatergic synapses in FS interneurons, while not affecting glutamatergic synapses in mPFC pyramidal neurons. At the mPFC neuronal network level, blockade of A_2A_R reduced LTP induction, suggesting a role for A_2A_R at the network level.

Target cell specificity of A_2A_R modulation has also been found in the hippocampus, although with the difference that activation of hippocampal A_2A_R increased excitatory transmission to CA1 pyramidal cells but not to inhibitory interneurons (Rombo et al., 2015). The PFC is unique in the magnitude and variety of interneurons, where FS interneurons represent the largest group (Markram et al., 2004). FS interneurons are activated by feedback and feedforward excitation, and they target perisomatic regions of pyramidal neurons (Tremblay et al., 2016) to control the output of pyramidal neurons by exerting fast, powerful and uniform inhibition of their firing (Kvitsiani et al., 2013; Sparta et al., 2014). Both LTP and LTD can be generated in FS interneurons, although LTP seems to be the dominant form of plasticity expressed in this neuron subtype (Lamsa et al., 2007; Lu et al., 2007; Sarihi et al., 2008; Nissen et al., 2010; Sambandan et al., 2010). In contrast to long-term plasticity (LTP) of excitatory synapses onto pyramidal neurons, LTP at glutamatergic synapses in FS neurons is predominantly independent of NMDA receptors (Lamsa et al., 2007; Sarihi et al., 2008; Nissen et al., 2010; Sambandan et al., 2010; Huang et al., 2013). In most cases, an essential role for group I metabotropic glutamate receptors (mGluRs) has been demonstrated in LTP and LTD induction in these FS interneurons (Perez et al., 2001; Lu et al., 2007; Sarihi et al., 2008; Huang et al., 2013). Whether LTP or LTD can be induced in these synapses is dependent on post-synaptic calcium fluctuations during LTP induction (Alle et al., 2001; Sambandan et al., 2010; Huang et al., 2013). A_2A_R control both NMDA receptors and voltage-sensitive calcium channels, thus potentially contributing to modulate the pattern of plasticity (Mogul et al., 1993; Gonçalves et al., 1997; Rebola et al., 2008; Azdad et al., 2009; Higley and Sabatini, 2010). Furthermore, A_2A_R heteromerize with mGluR5 (Ferré et al., 2002) and tightly interact with mGluR5 receptor function in the hippocampus, changing the efficiency of NMDA receptors (Tebano et al., 2005; Sarantis et al., 2015; Viana da Silva et al., 2016). Whether either of these mechanisms is responsible for the observed effects, should be subject to further investigation.

We here show the role of A_2A_R in normal, non-pathological, conditions by targeting the endogenous pool of adenosine acting at the A_2A_R with the A_2A_R antagonist SCH58261. In these conditions, A_2A_R act mainly as modulators of synaptic plasticity (d’Alcantara et al., 2001; Rebola et al., 2008; Simões et al., 2016). A_2A_R have an additional role in pathological conditions, where they can control microglia and astrocytes (Rebola et al., 2011; Matos et al., 2015; Orr et al., 2015; Cunha, 2016). Targeting A_2A_R with an A_2A_R agonist would mimic the situation of an additional load onto these microglia-and astrocytic located A_2A_R, thereby recruiting A_2A_R that are only active in pathological conditions (Matos et al., 2012; Orr et al., 2015). As we are specifically interested in the role of the A_2A_R in non-pathological conditions, we only evaluated plastic changes under influence of the A_2A_R antagonist.

The alteration in glutamatergic synapse strength in FS interneurons by A_2A_R can have a major impact on cortical function. A decreased synaptic strength at FS interneurons has been linked to a loss of temporal fidelity of pyramidal-to-pyramidal signaling (Lamsa et al., 2005), leading to a loss of information processing (Pouille and Scanziani, 2001). Also, the selective control by A_2A_R of plasticity at glutamatergic synapses onto FS interneurons might have important implications for the excitation–inhibition balance. Indeed, if the activity of interneurons is experimentally reduced in mPFC, LTP of excitatory to pyramidal neurons is impaired (Konstantoudaki et al., 2016). Adenosine, by acting at A_2A_R at synapses of FS interneurons, could therefore provide a homeostatic mechanism by which inhibition is ensured, thereby maintaining a proper excitation–inhibition balance (Zhang et al., 2015). FS interneurons, in particular the parvalbumin-positive FS cells, have been shown to support working memory and cognitive flexibility (Murray et al., 2015), and to be central for the control of attention (Kim et al., 2016). Therefore, abnormal A_2A_R function might lead to impaired behavioral functioning through changes in plasticity at FS interneuron synapses. An overexpression of A_2A_R specifically in the PFC is indeed related to cognitive and attentional deficits in a rat model of attention deficit and hyperactivity disorder (Pandolfo et al., 2013). Furthermore, genetic elimination of A_2A_R also interferes with behaviors that involve information processing in the PFC, including working memory (Zhou et al., 2009; Wei et al., 2011) and reversal learning (Wei et al., 2011). Future research targeting selectively A_2A_R in PFC FS interneurons will be needed to elucidate whether specifically A_2A_R located on glutamatergic synapses in FS interneurons control PFC-related behavior.

In short, we present here a first characterization of the role of endogenous adenosine acting at A_2A_R to affect synaptic plasticity in the mPFC, showing that A_2A_R specifically affect plasticity of glutamatergic synapses in cortical FS interneurons. An effect of A_2A_R manipulation on plasticity at these synapses was never shown before, therefore, further explorations into synaptic plasticity of FS interneurons in the PFC should be considered to reveal the underlying mechanism of A_2A_R manipulation.

## Author Contributions

AK, HM, RC, and SF designed the research. AK, SF, AT, TH, JR, CX, PC, and RC performed the experiments. AK, SF, RC, and HM analyzed the data. AK and SF wrote the first draft of the manuscript. All authors commented on the manuscript text.

## Conflict of Interest Statement

RC is a scientific consultant for the Institute for Scientific Information on Coffee. The other authors declare that the research was conducted in the absence of any commercial or financial relationships that could be construed as a potential conflict of interest.
